# The frog skin that saved fifty million lives — from the bench-side to world-wide 

**Published:** 2019

**Authors:** David Sachar, Norbert Hirschhorn

**Affiliations:** *Mount Sinai School of Medicine, New York, USA *

 Life-saving oral rehydration therapy (ORT) for dehydrating diarrhoea (caused by cholera and other intestinal infections, now including Ebola) had its first practical application fifty years ago, with upwards of fifty million adults and children saved since then (1). What made ORT compelling to consider back then, if it could be developed, was that in many parts of the world life-saving intravenous fluid and equipment were (still are) in short supply, with few persons at hand skilled in using them, especially in diarrhoea epidemics such as we see today in Yemen, Africa, and Haiti.

The road to its development seems in retrospect ordered and straight, but as in most scientific and medical breakthroughs, this was hardly the case (2). Moreover, such a remarkable medical treatment has many latter day claimants to its development and success. Nonetheless, certain individuals and experiments stand out. For instance, the 1950s-60s discovery of active absorption of glucose and sodium co-transported across the intestinal epithelium was hailed by The Lancet as “potentially the most important medical advance this century”, because it opened the way to use of ORT (3). The finding was necessary to the understanding of how ORT might work in cholera, but insufficient and needed clinical validation in the field as provided by Nalin and Cash (1). In fact, reigning theories about the pathology of cholera weighed against ORT: cholera thought due to destruction of the intestinal lining, leading to leakage of fluids; or cholera thought due to the “poisoning” of a theoretical intestinal sodium pump.

The first clear evidence for the prospect of ORT came from the work of Dr. David Sachar (DS) at the Cholera Research Laboratory (CRL) in East Pakistan (now the International Center for Diarrhoeal Disease Research, Bangladesh), sponsored by the National Institutes of Health and the US Centers for Disease Control. Here is where the frog skin comes in. 

In 1966, DS, on assignment as a U.S. Public Health Officer at the Cholera Research Laboratory in Dhaka, East Pakistan set out to explore the basic mechanisms of diarrhea in cholera, a leading killer of children and adults worldwide. Starting with experience measuring electric potentials across frog skins in Prof. Hans Ussing’s laboratory in Copenhagen, DS developed an ingenious but simple method for measuring electric potential in the intact human intestine. He imported this technique back to his base in Dhaka, where he could use it to test the function of patients’ intestinal sodium transport during the course of cholera. By assessing intestinal potential at the height of their diarrhea and again in convalescence, DS demonstrated not only that active sodium absorption was intact throughout the disease, but that it was also robustly stimulated by the infusion of glucose into the intestinal lumen. The finding, published in Gastroenterology in 1969 (4), gave confidence for Norbert Hirschhorn (NH), his colleague at the time, to conduct a proof-of-concept demonstration by that a glucose-sodium solution steadily infused into the intestines of cholera patients could actually reduce the secretions into the gut (5). In the following years ORT has become used world-wide, in dire circumstances, for all ages, administered by lightly-trained health workers or families, with the results well known. Its most dramatic success on a national scale was shown in Egypt when elements of manufacture, distribution, training of health workers, establishment of rehydration corners in clinics and hospitals, and use of television spots to educate families in the necessity and use of ORT led to dramatic reductions of infant and child mortality (6). 

## Conflict of interests

The authors declare that they have no conflict of interest.
